# Systematic review on the impact of exercise on intraocular pressure in glaucoma patients

**DOI:** 10.1007/s10792-024-03216-4

**Published:** 2024-08-19

**Authors:** D. González-Devesa, D. Suárez-Iglesias, J. C. Diz, A. Esmerode-Iglesias, C. Ayán

**Affiliations:** 1 Faculty of Physical Activity and Sports Sciences, Universidad de León, León, Spain; 2 VALFIS Research Group, Institute of Biomedicine (IBIOMED), Universidad de León, León, Spain; 3 Well-Move Research Group, Galicia Sur Health Research Institute (IIS Galicia Sur), SERGAS-UVIGO, Vigo, Spain; 4 Departamento de Didácticas Especiais, Universidade de Vigo, Vigo, Spain; 5 Facultad de Ciencias de la Educación y del Deporte, Universidad de Vigo, Pontevedra, España

**Keywords:** Aerobic training, Complementary therapy, Eye health, Resistance training, Vision preservation

## Abstract

Due to limited studies, we systematically reviewed evidence on the impact of physical exercise on intraocular pressure (IOP) in glaucoma patients, adhering to PRISMA guidelines. Using MEDLINE/Web of Science, PubMed, and Scopus, we selected English, Portuguese, or Spanish studies excluding case reports and yoga-based interventions. From 1001 records, 15 studies were independently evaluated. Evaluated through the MMAT scoring system, two quantitative randomised controlled studies scored 100% while 13 non-randomised studies averaged 84.62%. Our findings indicated that both aerobic and resistance training led to an immediate IOP reduction post-exercise. However, these findings were largely from single-session experiments. In contrast, the effects of longer-term exercise programmes on IOP varied. Although our review underscores the potential utility of exercise in IOP management, the evidence remains inconclusive due to variations in study design, participant demographics, and exercise parameters. This lack of consistency in the research highlights the necessity for larger, standardised, and longer-term studies to robustly corroborate these preliminary findings.

## Introduction

Glaucoma, an optic neuropathy with chronic progression, leads to irreversible vision loss on a global scale primarily due to ongoing damage to the optic nerve and retinal nerve fibre layer [1]. The main treatment strategy for glaucoma involves targeting the intraocular pressure (IOP), recognised as the single modifiable risk factor, through various means including medications, laser procedures, or surgery [2]. Additionally, recent studies show that lifestyle modifications, especially active and healthy living, can reduce IOP. Many patients have consulted their doctors for lifestyle guidance based on this evidence [3].

Physical activity is an important but often overlooked factor that affects glaucoma progression, according to recent research [4]. However, the scientific community is yet to reach a consensus regarding the effects of exercise on glaucoma. Some theories suggest that an exercise-induced IOP elevation may lead to reduced ocular perfusion pressure, possibly causing mechanical or ischaemic damage to the optic nerve head [5]. Contrarily, other studies propose that exercise can trigger a reduction in IOP levels, thus positively affecting ocular health [6].

Given this context of unclear evidence, the onus falls on ophthalmologists, general practitioners, and sports medicine specialists to provide well-informed, evidence-based guidance on physical activity to glaucoma patients. Achieving this requires the rigorous undertaking of systematic reviews that summarise and critically evaluate existing scientific evidence on the subject. Up to now, the only systematic review that specifically addresses the influence of exercise on glaucoma is centred solely around yoga [7]. To rectify this lack of comprehensive studies, we plan to carry out a systematic review with the aim of identifying and critically evaluating the most robust evidence available on the effects of physical exercise training programmes on IOP in glaucoma patients.

## Materials and methods

We adhered to the Preferred Reporting Items for Systematic Reviews and Meta-Analyses (PRISMA) guidelines [8]. We registered this review with the Open Science Framework (OSF), 10.17605/OSF.IO/MA9XF.

### Search strategy

A systematic search of three electronic databases (MEDLINE/Web of Science, PubMed, and Scopus) was undertaken from their inception until April 2023, employing a combination of the keywords “Glaucoma” AND “exercise”.

### Eligibility criteria

Research providing insights into the impacts of physical exercise on the IOP of individuals with glaucoma was deemed eligible. Studies were excluded based on the following criteria: (a) data derived from a case study; (b) intervention was yoga-based; (c) exercise was paired with other therapies; (d) the sample incorporated both healthy individuals and glaucoma patients with data not separately presented for each group; (e) IOP was not a target outcome; (f) unavailability of the study’s full-text; (g) research not authored in English, Portuguese or Spanish.

### Study selection

Two authors independently screened the titles and abstracts of identified studies for eligibility. After independently assessing the selected studies, these were compared by both authors to attain consensus, adhering to the inclusion criteria. Once consensus was established, the full-text of each potentially relevant study was procured. If it was ambiguous whether the study met the selection criteria, a third author was consulted and a consensus was reached, following the inclusion criteria. The reference lists of selected articles, as well as studies that cited them, were scrutinised for potentially new articles suitable for this review.

### Data extraction

Study type, participant characteristics, performed exercise interventions, measurement tool utilised for determining IOP, significant intra and inter-group changes in IOP post-intervention, and completion rate were retrieved from the original reports by one researcher and corroborated by a second investigator.

### Quality appraisal

Quality was evaluated employing the Mixed Methods Appraisal Tool (MMAT) [9]. The tool is applicable to quantitative, qualitative, and mixed methods primary studies. Papers were not excluded from the review based on low-quality scores, yet quality scores were reported and factored into the synthesis of the evidence. Scores are methodology-specific and are grounded in controlling confounding factors; completeness of outcome data; minimising selection bias; sample representativeness; appropriateness of measures; response and withdrawal rates; appropriateness of study design in answering the research questions; and consideration of limitations. After calculating specific percentages, the scores were divided into four categories: poor (0–25%), low (26–50%), medium (51–75%), and high (76–100%) [10]. Quality performance is delineated in Table 3 below.

### Statistical analysis

Meta-analysis computations were performed in Microsoft Excel with Meta-Essentials Workbooks [11], utilising the workbook of dependent groups-continuous data, in line with the format of the collected data. A random effects model was used for all analyses to account for expected sources of heterogeneity among different studies. I^2^ was employed to evaluate statistical heterogeneity and inconsistency, with I^2^ values of 0% denoting no observed heterogeneity, and higher values indicating increased heterogeneity.

A forest plot was used to summarise the findings, adopting Hedges’ g test as a measure of exercise's effect size on IOP. Funnel plot and Egger’s test were utilised to statistically assess the presence of any publication bias. The trim-and-fill analysis was also incorporated for the adjustment of potentially missing studies. We also carried out a moderator analysis of the effect of basal IOP and exercise intensity on effect size.

## Results

**Fig. 1:**
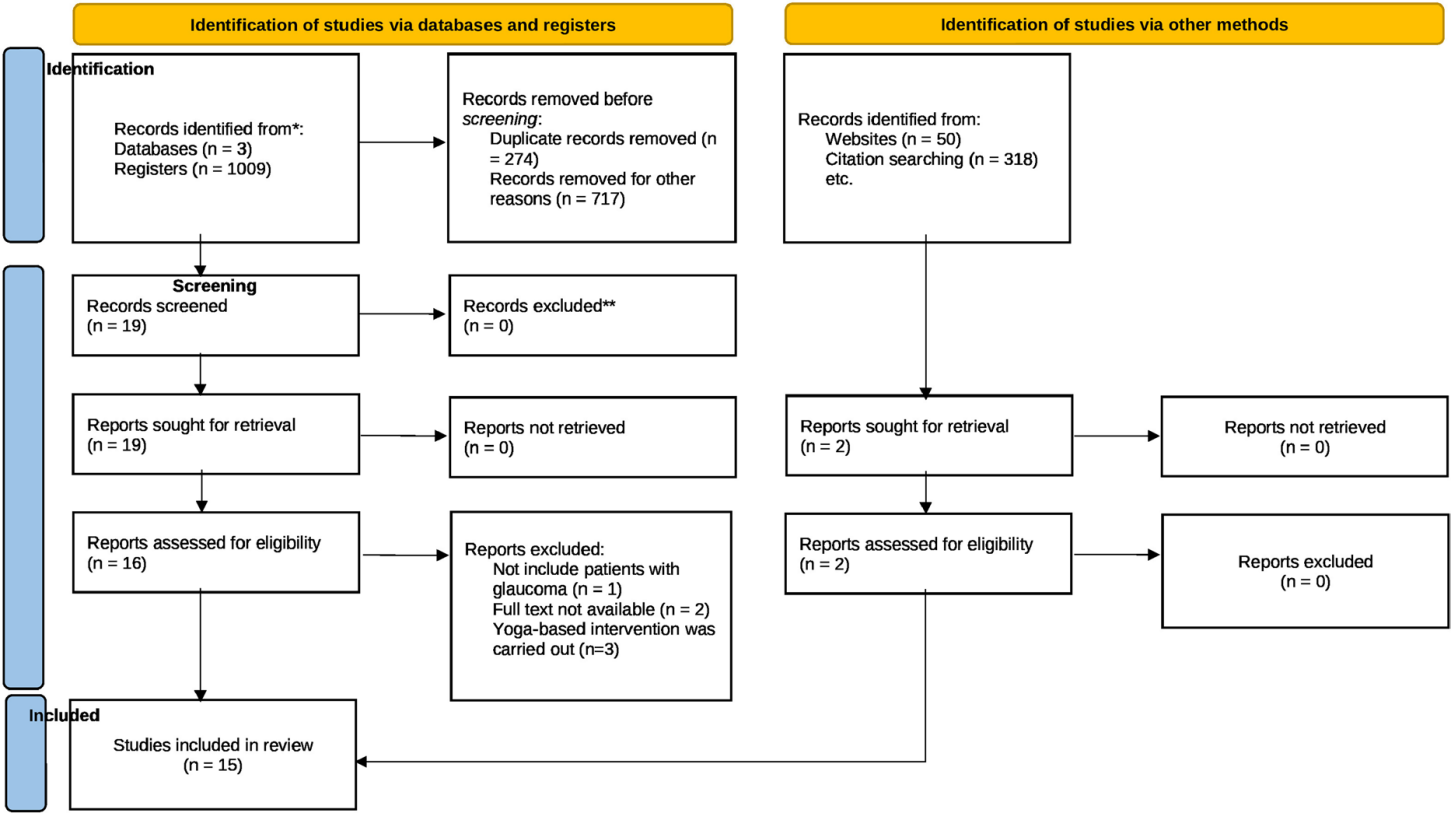
PRISMA (Preferred Reporting Items for Systematic Reviews and Meta-Analyses) study flow diagram

Our database search yielded 1001 records. After removing duplicates, we assessed the titles and abstracts of 727 records. We then conducted a full-text examination of 19 articles, ultimately identifying 15 studies that met our inclusion criteria for systematic review (Fig. 1).

### Design and samples

Among the 15 studies in our analysis, 11 of these [12–22] explored the impact of a single exercise session. The four remaining studies comprised two randomised controlled trials (RCTs) [23, 24], a comparative study [25], and a single group study [26] that implemented an exercise programme.

Together, these studies encompassed a total of 728 participants. The smallest study featured a sample of six participants [26], while the largest included 145 participants [16]. Except for one study [20], all studies reported the sex of participants, which accounted for 387 males and 329 females. Participants’ ages across the studies ranged from 13 to 78 years.

The Goldmann-Applanation Tonometer emerged as the primary measurement tool utilised in the selected studies [12–14, 16–25]. Furthermore, one study [21] employed a non-contact pneumatonometer (NCT, Canon HY9-RK-F1 Japan, automatic mode), another study [15] used the Perkins hand-held applanation tonometer, and Lipkova et al. [25] utilised Schiotz’s tonometer. However, Lipková & Kyselovičová [26] did not disclose the measurement tool used in their study.

### Interventions characteristics

Specifics about the interventions’ characteristics are provided in Tables 1 and 2. Out of the studies proposing a singular exercise session, aerobic activity was the most prevalent intervention (n = 10). Notably, resistance training was only evaluated in one study [18], and another study [22] analysed the effects of the isometric training. Regarding exercise programmes, three proposed aerobic exercises [23, 25, 26], while a solitary study analysed a programme based on resistance exercises [24].

**Table 1:** Overview of the studies proposing a training programme

First author (Year), design and participants	Intervention and IOP measurement tool	Significant effects
**Ma et al. (2022)** ** Design**: RCT **Participants (n; gender):** 123 (100); 62M + 61F *Age, years (median; mean; IQR; SD; range):* IG: 52 (49.83), 42–58 (1.5); 21–70; CON: 47 (47.48); 39–57 (1.6); 22–67 **Dropout rate (n; reasons):** 3 dropped out due to unavailability for follow-up, and 23 were excluded from the analysis of long-term effects (3 months) due to poor quality exercises	**Duration:** 3 months **IG:** Aerobic group **Activity description:** Regular jogging for four hours **Frequency:** 20 days/month **Volume:** 30 min **Intensity:** NR **CON:** Irregular exercises ***Measurement tool:*** Goldmann-Applanation Tonometer	**Intra-group (*p* < 0.05)** ↓ IOP in *IG* after exercise ↑ IOP in *IG* after 3 months **Inter-group (*p* < 0.05)** ↑ IOP in *IG* than in *CON* after 3 months
**Ibrahim et al. (2019)** **Design**: RCT **Participants (n; gender):** 27; 17M + 10F *Age, years (mean; SD):* IG: 46.93 ± 3.17; CON: 48.2 ± 2.68 **Dropout rate (n; reasons):** 3 patients due to illnesses or withdrawal	**Duration:** 4 weeks **IG:** Resistance training + standard medical treatment **Activity description:** Warm-up: 5–10 min, stretching + main phase: 20 min, resistance exercises for the upper limb (3 × 8 rep with 2 min rest between sets) + cool-down: 5 min, stretching **Frequency:** 3 days/week **Volume:** 30–35 min **Intensity:** 40–60% 1RM of biceps brachii **CON:** Standard medical treatment ***Measurement tool:*** Goldmann-Applanation Tonometer	**Intra-group (*p* < 0.05)** ↓ IOP-r in *IG* (20.27 ± 3.62 vs. 17.53 ± 3.31 mmHg) ↓ IOP-l in *IG* (21.87 ± 2.48 vs. 16.6 ± 2.38 mmHg) **Inter-group (*p* < 0.05)** > IOP-r in *CON* than in *IG* (20.87 ± 1.06 vs. 17.53 ± 3.31 mmHg) > IOP-l in *CON* than in *IG* (20.27 ± 1.71 vs. 16.6 ± 2.38 mmHg)
**Lipkova et al. (2008)** **Design**: Comparative **Participants (n; gender):** 15F *Age, years (mean; SD):* IG: 49.4 ± 2.2; CON: 48.9 ± 2.3 **Dropout rate:** NR	**Duration**: 8 weeks **IG:** Aerobic group **Activity description:** Warm–up: 10–15 min low impact aerobics + pre–stretching; main aerobics part: 20–30 min of aerobics movements done continuously or in intervals within aerobic training zones of individuals; cool down: 5 min of slow relaxation and stretches **Frequency:** 3 days/week **Volume:** 45 min **Intensity:** 50–85% HR_max_ **CON:** Standard medical treatment ***Measurement tool:*** Goldmann’s and Schiotz’s tonometer	**Intra-group (*p* < 0.05)** ↑ IOP in *IG* only in one session during intervention
**Lipkova et al. (2011)** **Design**: Single group **Participants (n; gender):** 6F *Age, years (mean; SD):* 54 ± 3.4 **Dropout rate:** NR	**Duration:** 3 months **IG:** Aerobic group **Activity description:** Aerobic dance and fitball aerobic exercises were alternated. Warm–up: 10–15 min low impact aerobics + pre–stretching; main aerobics part: 20–30 min of aerobics continuous, alternating load; cool down: 5–7 min of static stretching exercises **Frequency:** 2 days/week **Volume:** 55–60 min **Intensity:** 50–85% HR_max_ ***Measurement tool:*** NR	No significant effect found

> , Greater; < , Lower; ↑, Increment; ↓, Decrement; 1RM, one repetition maximum; Δt represents gain score i.e., the change in the value in comparison to the baseline; CON, Control Group; DBP, Diastolic Blood Pressure; F, Female; HR, Heart Rate; IG, Intervention Group; IOP, Intra‑Ocular Pressure; IOP-l, IOP of Left; IOP-r, IOP of Right Eye; M, Male; NR, Not Reported; RCT, Randomised Controlled Trial; SBP, Systolic Blood Pressure; W_max_, Maximum Exercise Power, in Watt

**Table 2:** Characteristics of studies involving a single exercise session

First author (Year), design and Participants	Intervention and IOP measurement tool	Significant effects
**Nie et al. (2022)** ** Design**: Comparative **Participants (n; gender):** 47; IG: 25 with glaucoma (16 M + 9F); CON: 22 healthy (14M + 8F) *Age, years (mean; SD; range):* IG: 32 ± 6.18, 23–45; CON: 30.91 ± 5.66, 25–43 **Dropout rate:** 0	**Aerobic exercise** **Activity description:** Continuous running on a treadmill. **Volume:** 20 min **Intensity:** 6–8 km/h to simulate moderate intensity ***Measurement tool:*** Goldmann-Applanation Tonometer	**Intra-group (*p* < 0.05)** ↓ IOP in *IG* immediately after exercise (16.52 ± 3.35 vs. 14.2 ± 2.51 mmHg) ↑ IOP in *IG* at 30 min after exercise compared to immediately after exercise (15.84 ± 3.57 vs. 14.2 ± 2.51 mmHg) ↓ IOP in *CON* immediately after exercise (16.01 ± 2.42 vs. 14.51 ± 2.96 mmHg) ↑ IOP in *CON* at 30 min after exercise compared to immediately after exercise (16.5 ± 2.25 vs. 14.51 ± 2.96 mmHg)
**Cheng et al. (2022)** **Design**: Comparative **Participants (n; gender):** 39; IG: 19 with glaucoma (15M + 4F); CON: 20 healthy (13M + 7F) *Age, years (mean; SD):* IG: 31.47 ± 6.36, CON: 30.95 ± 5.80 **Dropout rate:** 0	**Aerobic exercise** **Activity description:** Continuous running on a treadmill. **Volume:** 20 min **Intensity:** 6–8 km/h to simulate moderate intensity ***Measurement tool:*** Goldmann-Applanation Tonometer	**Intra-group (*p* < 0.05)** ↓ IOP in *IG* immediately after exercise (16.81 ± 3.22 vs. 14.21 ± 2.48 mmHg) ↑ IOP in *IG* at 30 min after exercise compared to immediately after exercise (16.32 ± 3.62 vs. 14.21 ± 2.48 mmHg) ↓ IOP in *CON* immediately after exercise (16 ± 2.43 vs. 14.6 ± 3.05 mmHg) ↑ IOP in *CON* at 30 min after exercise compared to immediately after exercise (16.52 ± 2.37 vs. 14.6 ± 3.05 mmHg)
**Yuan et al. (2021)** **Design**: Comparative **Participants (n; gender):** 71; IG: 35 with glaucoma (31M + 4F); CON: 36 healthy (15M + 21F) *Age, years (mean; SD):* IG: 36.09 ± 10.53; CON: 31.97 ± 9.65 **Dropout rate:** NR	**Aerobic exercise** **Activity description:** Continuous running on a treadmill. **Volume:** 30 min **Intensity:** Moderate, 60–80% HR_max_ ***Measurement tool:*** Goldmann-Applanation Tonometer	**Intra-group (*p* < 0.05)** ↓ IOP in *IG* after exercise (16.82 ± 4.4 vs. 14.7 ± 3.52 mmHg) ↓ IOP in *CON* after exercise (13.04 ± 2.31 vs. 12.03 ± 1.73 mmHg) **Inter-group (*p* < 0.05)** > IOP decrease in *IG* than in *CON* after exercise (‑2.12 ± 0.25 vs. − 1.04 ± 0.22 mmHg)
**Umoh et al. (2020)** **Design**: Comparative **Participants (n; gender):** 40; IG: 20 with glaucoma (14M + 6F); CON: 20 healthy (11M + 9F) *Age, years (mean; SD):* IG: 49.4 ± 8.4; CON: 43.8 ± 10.1 **Dropout rate:** 0	**Aerobic exercise** **Activity description:** Jogged on a treadmill (inclined at 10 degrees) **Volume:** 7 min **Intensity:** 2 miles/hour ***Measurement tool:*** Perkins hand held applanation tonometer	**Intra-group (*p* < 0.05)** ↓ IOP-r in *IG* immediately after exercise (18 ± 5.7 vs. 16.2 ± 4.9 mmHg) ↓ IOP-r in *IG* at 60 min after exercise (18 ± 5.7 vs. 16.6 ± 4.7 mmHg) ↓ IOP-r in *CON* immediately after exercise (14.8 ± 1.6 vs. 12.6 ± 1.6 mmHg) ↓ IOP-l in *IG* immediately after exercise (18.4 ± 6.1 vs. 16.3 ± 4.9 mmHg) ↓ IOP-l in *IG* at 60 min after exercise (18.4 ± 6.1 vs. 17.3 ± 5.4 mmHg) ↓ IOP-l in *CON* immediately after exercise (15.1 ± 2.5 vs. 13 ± 2.1 mmHg) **Inter-group (*p* < 0.05)** > IOP-r in *IG* than in *CON* at baseline (18 ± 5.7 vs. 14.8 ± 1.6 mmHg) > IOP-r in *IG* than in *CON* immediately after exercise (16.2 ± 4.9 vs. 12.6 ± 1.6 mmHg) > IOP-r in *IG* than in *CON* at 5 min after exercise (15.6 ± 4.6 vs. 12.7 ± 2.1 mmHg) > IOP-r in *IG* than in *CON* at 10 min after exercise (16.3 ± 4.9 vs. 12.8 ± 1.9 mmHg) > IOP-r in *IG* than in *CON* at 30 min after exercise (16.1 ± 4.7 vs. 12.3 ± 1.7 mmHg) > IOP-r in *IG* than in *CON* at 60 min after exercise (16.6 ± 4.7 vs. 12.9 ± 1.8 mmHg) > IOP-l in *IG* than in *CON* at baseline (18.4 ± 6 vs. 15.1 ± 2.5 mmHg) > IOP-l in *IG* than in *CON* immediately after exercise (16.3 ± 4.9 vs. 13 ± 2.1 mmHg) > IOP-l in *IG* than in *CON* at 5 min after exercise (16.8 ± 5.4 vs. 13.4 ± 2.1 mmHg) > IOP-l in *IG* than in *CON* at 10 min after exercise (16.5 ± 5.6 vs. 13.3 ± 1.8 mmHg) > IOP-l in *IG* than in *CON* at 30 min after exercise (16.8 ± 5.3 vs. 13 ± 2.2 mmHg) > IOP-l in *IG* than in *CON* at 60 min after exercise (17.3 ± 5.4 vs. 13 ± 2.1 mmHg)
**Natsis et al. (2009)** **Design**: Comparative **Participants (n; gender):** 145; IG1: 40 normotensive b-blocker in RE (24 M + 16F); IG2: 20 normotensive prostaglandin analogue in RE (18 M + 2F); IG3: 15 normotensive a-agonist in RE (10 M + 5 F); IG4: 15 primary glaucoma under b-blockers (8 M + 7F); IG5: 15 primary glaucoma under prostaglandin analogues (7 M + 8F); IG6: 15 primary glaucoma under combination of anti-glaucoma drugs (5 M + 10F); CON: 25 normotensive no medication was instilled (16 M + 9F) *Age, years (mean; range):* IG: 35.9, 20–51; IG2: 36.7, 27–55; IG3: 28.7, 17–38; IG4: 62.5, 47–78; IG5: 61, 50–75; IG6: 64, 50–78; CON: 15.5, 13–18 **Dropout rate:** NR	**Aerobic exercise** **Activity description**: Cycle-ergometer **Volume:** 10 min **Intensity:** 60–80 Watts ***Measurement tool:*** Goldmann-Applanation Tonometer	**Intra-group (*p* < 0.05)** ↓ IOP-r in *CON* after exercise (13.36 ± 1.79 vs. 10.6 ± 2.24 mmHg) ↓ IOP-r in *IG1* after exercise (15.75 ± 1.46 vs. 9.8 ± 1.36 mmHg) ↓ IOP-r in *IG2* after exercise (14.08 ± 1.78 vs. 9.25 ± 1.9 mmHg) ↓ IOP-r in *IG3* after exercise (14.66 ± 2.38 vs. 7.4 ± 2.02 mmHg) ↓ IOP-r in *IG4* after exercise (17.13 ± 2.39 vs. 14.53 ± 2.35 mmHg) ↓ IOP-r in *IG5* after exercise (15.93 ± 2.31 vs. 13.6 ± 1.8 mmHg) ↓ IOP-r in *IG6* after exercise (16.73 ± 2.34 vs. 14.27 ± 1.79 mmHg) ↓ IOP-l in *CON* after exercise (13.16 ± 1.54 vs. 10.58 ± 2.11 mmHg) ↓ IOP-l in *IG1* after exercise (15.62 ± 1.37 vs. 11.65 ± 1.51 mmHg) ↓ IOP-l in *IG2* after exercise (14.35 ± 2.1 vs. 12.13 ± 1.58 mmHg) ↓ IOP-l in *IG3* after exercise (14.06 ± 2.08 vs. 10.4 ± 2.55 mmHg) ↓ IOP-l in *IG4* after exercise (16.27 ± 2.31 vs. 13.73 ± 2.31 mmHg) ↓ IOP-l in *IG5* after exercise (15.6 ± 2.47 vs. 13.13 ± 2.45 mmHg) ↓ IOP-l in *IG6* after exercise (18.5 ± 3.04 vs. 15.67 ± 2.61 mmHg)
**Qureshi (1995)** **Design**: Comparative **Participants (n; gender):** 14; IG: 7 with glaucoma, NR; CON: 7 M healthy *Age, years (mean; SD; range):* IG: 46.14 ± 3.23, 40–50; CON: 44.42 ± 3.15, 40–50 **Dropout rate:** NR	**Aerobic exercise** **Activity description:** Walking 1 h + jogging 1 h + running as fast as possible until volitional exhaustion. **Volume:** ~ 2:10 h **Intensity:** NR ***Measurement tool:*** Goldmann-Applanation Tonometer	**Intra-group (*p* < 0.05)** ↓ IOP in *IG* at 5 min of walking (33.43 ± 2.19 vs. 29.14 ± 1.77 mmHg) ↓ IOP in *IG* at 20 min of walking (33.43 ± 2.19 vs. 27.43 ± 1.53 mmHg) ↓ IOP in *IG* at 40 min of walking (33.43 ± 2.19 vs. 25.71 ± 1.23 mmHg) ↓ IOP in *IG* at 5 min of jogging (33.29 ± 2.24 vs. 26.71 ± 1.04 mmHg) ↓ IOP in *IG* at 20 min of jogging (33.29 ± 2.24 vs. 25.71 ± 0.87 mmHg) ↓ IOP in *IG* after running (32.86 ± 2.13 vs. 20 ± 0.82 mmHg) ↓ IOP in *CON* at 5 min of walking (15.29 ± 0.81 vs. 13.86 ± 0.8 mmHg) ↓ IOP in *CON* at 5 min of jogging (15.14 ± 0.74 vs. 12.86 ± 0.74 mmHg) ↓ IOP in *IG* at 40 min of jogging (15.14 ± 0.74 vs. 11.29 ± 0.57 mmHg) ↓ IOP in *CON* after running (15.14 ± 1.01 vs. 11.14 ± 0.86 mmHg)
**Gillmann et al. (2021)** **Design**: Prospective Single-Center Study **Participants (n; gender):** 14 (8 M + 6F) *Age, years (mean; SD):* 57.9 ± 13 **Dropout rate:** NR	**IG1: Aerobic exercise** **Activity description:** Walking and cycling **Volume:** NR **Intensity:** NR **IG2: Resistance training** **Activity description:** NR **Volume:** NR **Intensity:** NR **IG3: Yoga and meditation** **Activity description:** NR **Volume:** NR **Intensity:** NR **IG4: Emotional stress** **Activity description:** NR **Volume:** NR **Intensity:** NR **IG5: Alcohol consumption** **Activity description:** NR **Volume:** NR **Intensity:** NR ***Measurement tool:*** Goldmann-Applanation Tonometer	**Intra-group (*p* < 0.05)** ↑ IOP in *IG1* during activity ↑ IOP in *IG2* from activity onset to 120 min after activity ↑ IOP in *IG4* from stressful stimulus initiation to 120 min after stimulus cessation. ↓ IOP in *IG5* at the time of consumption
**Gracitelli et al. (2020)** **Design**: Single-group **Participants (n; gender):** 30 (16 M + 14F) *Age, years (mean; SD):* 62.9 ± 1.7 **Dropout rate:** 0	**Aerobic exercise** **Activity description:** Cycle-ergometer **Volume:** 40 min **Intensity:** Moderate, 70% HR_max_ ***Measurement tool:*** Goldmann-Applanation Tonometer	**Intra-group (*p* < 0.05)** ↑ IOP in *IG* after exercise and persisting higher after 30 min (11.5 ± 0.9 vs. 13.5 ± 0.9 and 12.3 ± 0.9 mmHg, respectively)
**Shapiro et al. (1983)** **Design**: Single-group **Participants (n; gender):** 12, NR *Age, years (range):* 40–69 **Dropout rate:** NR	**Aerobic exercise** **Activity description:** Progressive workload on the cycle ergometer at 50 cycles/min (3 × 5 min with 3 min rest between stages) **Volume:** 24 min **Intensity:** 0–75W (increased 25W at each stage) ***Measurement tool:*** Goldmann-Applanation Tonometer	**Intra-group (*p* < 0.05)** ↓ IOP in *IG* after 8 min of exercise (28.2 ± 9.4 vs. 24 ± 8.8 mmHg) ↓ IOP in *IG* after 16 min of exercise (28.2 ± 9.4 vs. 20.7 ± 8.8 mmHg) ↓ IOP in *IG* after 24 min of exercise (28.2 ± 9.4 vs. 19.9 ± 9.3 mmHg)
**Yang et al. (2014)** **Design**: Comparative **Participants (n; gender):** 80; IG: 30 glaucoma combined with high myopia (19M + 11F); IG2: 29 glaucoma combined with non-high myopia (12M + 17F); IG3: 21 glaucoma combined with non-myopia (9M + 12F) *Age, years (mean; SD):* IG: 45.17 ± 14.66; IG2: 50.10 ± 10.86; IG3: 55.71 ± 11.03 **Dropout rate:** 0	**Aerobic exercise** **Activity description:** 10 min of cycle ergometer + 2 min rest + 5 min of cycle ergometer **Volume:** 17 min **Intensity:** 20–60% W_max_ ***Measurement tool:*** Non-contact pneumatonometer (NCT, Canon HY9-RK-F1 Japan, automatic mode) and Goldmann-Applanation Tonometer	**Inter-group (*p* < 0.05)** > IOP decrease in *IG* versus *IG2* and versus *IG3,* after exercise (7.57 ± 3.47 vs. 4.66 ± 2.64 and 4.90 ± 3.37, respectively)
**Bata et al. (2019)** **Design**: Comparative **Participants (n; gender):** 65; IG: 32 with glaucoma (14M + 26F); CON: 33 healthy (14M + 26F) *Age, years (mean; SD):* IG: 58.9 ± 12.2; CON: 58.8 ± 12.5 **Dropout rate (n; reasons):** 15 not met the laser Doppler flowmetry reproducibility criterion	**Isometric exercise** **Activity description:** rest 20 min + handgrip 2 min + rest 2 min + handgrip 2 min + rest 2 min + handgrip 2 min + rest **Volume:** 30 min **Intensity:** 75% of individual maximal voluntary contraction ***Measurement tool:*** Goldmann-Applanation Tonometer	No significant effect found

> , Greater; < , Lower; ↑, Increment; ↓, Decrement; CON, Control Group; DBP, Diastolic Blood Pressure; F, Female; HR, Heart Rate; IG, Intervention Group; IOP, Intra‑Ocular Pressure; IOP-l, IOP of Left Eye; IOP-r, IOP of Right Eye; M, Male; NR, Not Reported; RCT, Randomised Controlled Trial; RE, Right Eye; SBP, Systolic Blood Pressure; W_max_, Maximum Exercise Power, in Watt

Four studies introduced training programmes of variable durations, from a concise 4-week programme [24] to an extended 3-month programme [23, 26]. These studies entailed 2–5 exercise sessions per week, with individual session durations ranging from 30 minutes [23] to 60 minutes [26]. Exercise intensity was typically regulated by maximal heart rate [25, 26] or by one repetition maximum, as in the case of Ibrahim & Elbeltagi [24]. However, Ma et al. [23] omitted details regarding exercise intensity.

### Dropouts and adverse events

From the six studies providing information about dropouts, a total of 44 were reported [22–24]. Dropout reasons were diverse, encompassing illness, study withdrawal, failure to meet reproducibility criteria, unavailability for follow-up, and subpar exercise performance. Nevertheless, none of the studies notified adverse effects ensuing from the interventions.

### Main outcomes

#### Single session studies

Ten out of the 11 studies that employed a single exercise session reported significant effects. Five of these studies displayed a significant intra-group reduction in IOP immediately following the aerobic exercise session [12–16]. In contrast, the study by Gracitelli et al. [19] demonstrated an elevated IOP after the intervention, which persisted for at least 30 minutes. Gillmann et al. [18] found an IOP increase during aerobic activity, lasting from the activity commencement to 120 minutes post-resistance exercise. Notably, IOP was higher 30 minutes after aerobic exercise than immediately post-exercise in three studies [12, 13, 19]. However, others documented a reduction in IOP at 24 minutes [20] and 60 minutes [15] after the intervention. No significant differences in IOP values were found in the sole study implementing isometric exercise [22].

Two out of the 11 studies revealed significant results in inter-group analysis [14, 15, 21]. Yuan et al. [14] showed a more substantial IOP decrease in glaucoma participants compared to healthy participants post-aerobic exercise. Additionally, Umoh et al. [15] reported consistently higher IOP levels in participants with glaucoma across all time points compared to healthy participants. In another study, participants with glaucoma and high myopia exhibited a more significant IOP reduction after aerobic exercise compared to both healthy participants and those with moderate myopia [21].

**Fig. 2:**
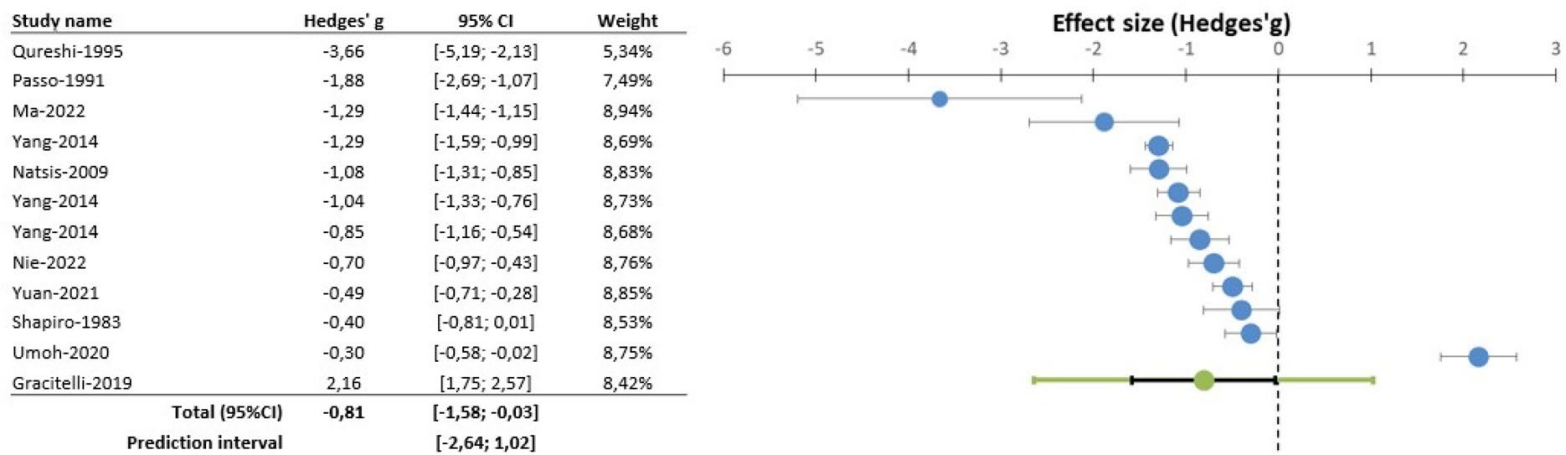
Meta-analysis of single exercise session impact on intraocular pressure

**Fig. 3:**
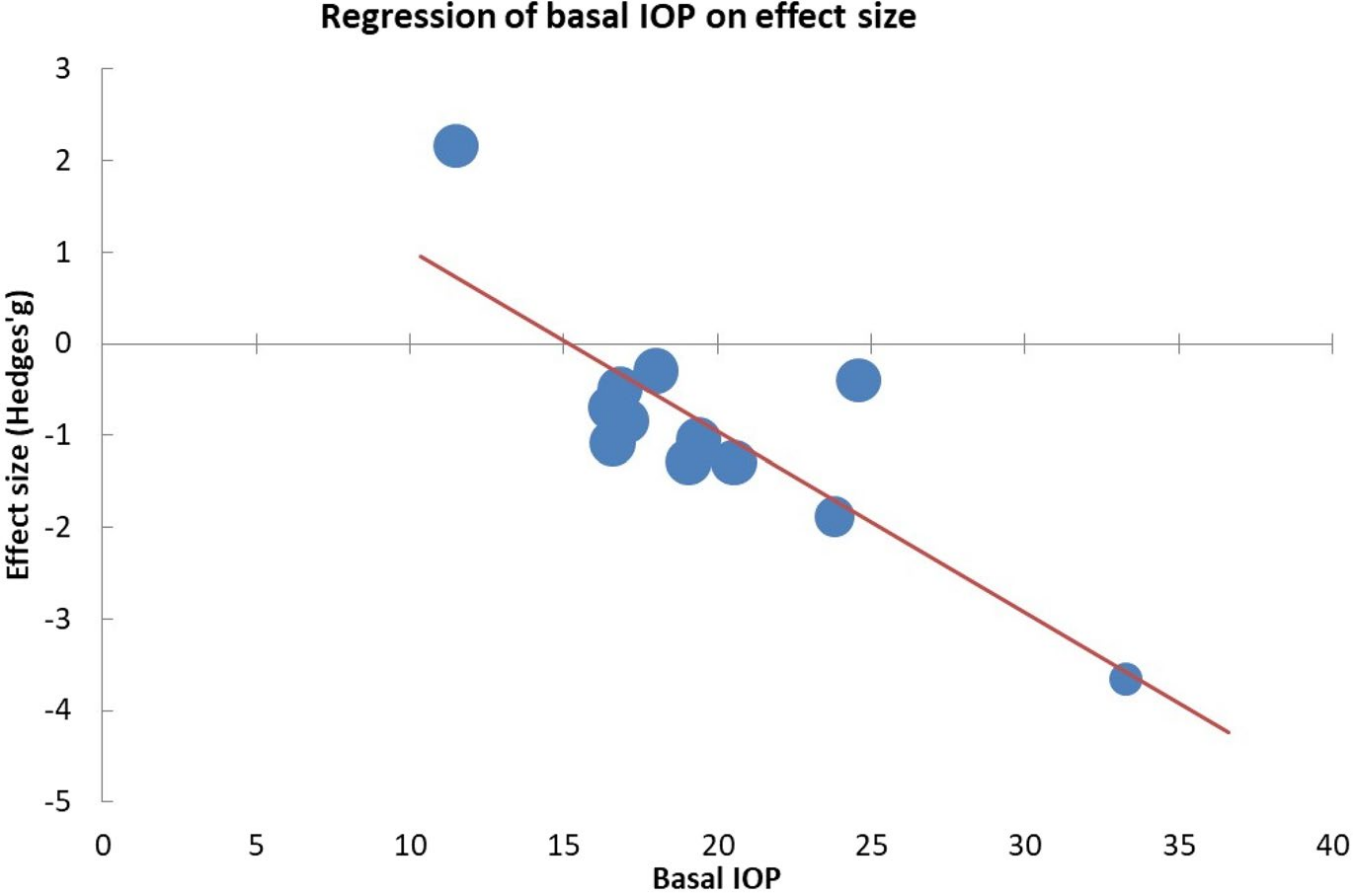
Regression of basal intraocular pressure (IOP) on effect size

Meta-analysis of the 12 studies (comprising 388 participants) comparing pre- versus post-intervention IOP mean values from single exercise sessions [12–22] indicated a moderately significant reduction in this outcome (Hedges’ g -0.81 (− 1.58; − 0.03) *p* = 0.022, albeit with high heterogeneity (I^2^ = 96.7%) (Fig. 2). Our results further suggested a strong dependence of exercise effects on baseline IOP: higher baseline IOP corresponded to more substantial exercise-induced reductions (Beta = − 0.20; *p* < 0.001; R^2^ = 0.626) (Fig. 3). Subsequent analysis of 24 subgroups (n = 701) showed that the IOP-lowering impact of exercise was independent of exercise intensity (Beta = 0.02; *p* = 0.889; R^2^ = 0.0003).

#### Exercise programme studies

Regarding the effects of exercise programmes, three of the four studies that implemented an intervention programme reported significant effects [23–25]. In all three studies, significant intra-group differences were observed. Ma et al. [23] reported a significant reduction in IOP values immediately after the aerobic intervention, but at 3 months post-intervention, IOP increased significantly in the aerobic group compared with irregular exercises group. In addition, Ibrahim and Elbeltagi [24] found significant improvements in IOP results following a resistance exercise-based intervention combined with standard medication. However, Lipkova et al. [25] only detected a significant increase of IOP in one session during the intervention.

Furthermore, only two of the four studies showed significant results in inter-group analysis [23, 24]. According to their findings, IOP tended to be higher in exercise protocols compared to the control group after the interventions.

### Quality appraisal

Overall, all 15 studies fulfilled most of the criteria outlined by the MMAT for each study design. The two quantitative randomised controlled studies [23, 24] had a MMAT score of 100% and the 13 quantitative non-randomised studies had a mean score of 84.62% (see Table 3).

**Table 3:** Methodological quality of the included studies according to the Mixed Methods Appraisal Tool (MMAT)

Quantitative randomised controlled trials						
	1. Is randomization appropriately performed?	2. Are the groups comparable at baseline?	3. Are there complete outcome data?	4. Are outcome assessors blinded to the intervention provided?	5. Did the participants adhere to the assigned intervention?	Total score %
Ma et al. [23]	Yes	Yes	Yes	Yes	Yes	100
Ibrahim et al. [24]	Yes	Yes	Yes	Yes	Yes	100

Quantitative non-randomised studies						
	1. Are the participants representative of the target population?	2. Are measurements appropriate regarding both the outcome and intervention (or exposure)?	3. Are there complete outcome data?	4. Are the confounders accounted for in the design and analysis?	5. During the study period, is the intervention administered (or exposure occurred) as intended?	Total score %
Lipkova et al. [25]	No	Yes	Can't tell	Yes	Can't tell	40
Lipkova et al. [26]	No	No	Can't tell	Yes	Can't tell	20
Nie et al. [12]	Yes	Yes	Yes	Yes	Yes	100
Cheng et al. [13]	Yes	Yes	Yes	Yes	Yes	100
Yuan et al. [14]	Yes	Yes	Yes	Yes	Yes	100
Umoh et al. [15]	No	Yes	Yes	Yes	Yes	80
Natsis et al. [16]	Yes	Yes	Yes	Yes	Yes	100
Qureshi [17]	Yes	Yes	Yes	Yes	Yes	100
Gillmann et al. [18]	No	Yes	Yes	Yes	Yes	80
Gracitelli et al. [19]	Yes	Yes	Yes	Yes	Yes	100
Shapiro et al. [20]	No	Yes	Yes	Yes	Yes	80
Yang et al. [21]	Yes	Yes	Yes	Yes	Yes	100
Bata et al. [22]	Yes	Yes	Yes	Yes	Yes	100

## Discussion

This research endeavoured to scrutinise and critically assess the highest quality evidence available regarding the influence of physical exercise on IOP in glaucoma patients. In total, we appraised 15 studies related to this subject and subsequently conducted a meta-analysis. Several findings from these studies warrant detailed discussion, given their potential significance for health professionals and exercise specialists.

Our findings suggest that specific forms of exercise, notably aerobic and resistance training, can induce an immediate post-exercise decrease in IOP, in both isolated sessions and ongoing exercise regimens. Nonetheless, it is critical to recognise that a majority of the studies under review were single-session experiments, and only two met the rigorous criteria of RCTs.

The meta-analysis we performed indicated an immediate post-exercise reduction in IOP among glaucoma patients, a finding that aligns with results from previous studies involving other populations. For instance, Conte et al. [27] found that both high-intensity interval training and continuous moderate exercise were efficacious in reducing IOP immediately post-exercise in healthy subjects. Similarly, Vera, Jiménez, Redondo, Cárdenas, et al. [28] demonstrated significant IOP reductions following two high-intensity interval exercise protocols in physically active collegiate individuals.

On the contrary, Risner et al. [29] showed a decrease in IOP after dynamic exercise, whereas the influence of isometric exercise on IOP remained more contentious. The single study within this review that explored the effects of an isometric exercise protocol did not report any significant impact on IOP [22]. Echoing these findings, Vera, Jiménez et al. [30] observed a swift return of IOP to baseline levels approximately 10 seconds post-isometric exercises.

The current review suggests that exercise programmes could exert a beneficial impact on IOP. However, there is a scarcity of studies investigating the effects of such interventions on IOP in either glaucoma patients or healthy subjects, thus limiting further discussion. Nevertheless, Yeak et al. [31] pointed out that a regular physical exercise programme, comprising aerobic and strength exercises, significantly lowered IOP in healthy subjects. Along similar lines, aerobic programmes have proven effective in reducing daytime blood pressure values [32]. Moreover, the meta-analysis conducted by Cornelissen et al. [33] suggested that a programme involving resistance exercises and isometric handgrip training could potentially reduce blood pressure. Due to data heterogeneity and unreliability, we could not perform a meta-analysis on the effect of exercise programmes on glaucoma patients, indicating a need for further research in this area.

Across the studies reviewed, a consistent reduction in IOP was reported, independent of the participants’ age or gender. Our findings align with the study by Vera et al. [28], which affirmed that IOP changes were not contingent on the participants’ sex.

In contrast, our findings regarding the effects of exercise intensity on IOP were somewhat unexpected. The meta-analysis suggested that the impact of exercise on lowering IOP was not dependent on the intensity of the exercise performed. This result contradicts previous research stating that to effectively reduce IOP, exercise intensity should exceed 70% of an individual’s maximum oxygen consumption [34].

Elevated IOP is a key risk factor in the onset and progression of glaucoma [4]. Prior studies have identified that sustained muscle contraction can cause an IOP increase [35], whereas relaxation is linked to a decrease [36]. Changes in body position also induce IOP variations [37], such as an elevation in head-down postures [7] or in supine positions as opposed to seated positions [38]. Furthermore, individuals with ocular hypertension may experience significant IOP increases in response to shifts in body position [39]. Therefore, glaucoma patients should avoid exercises that induce an IOP increase, particularly those associated with breath-holding [40]. Despite no adverse effects being detected from exercise interventions in this systematic review, given the potential risks posed by certain exercise types like resistance training [36], or activities such as swimming that encompass factors such as deep respiration, body position, and muscle effort [5], further exploration is necessary to ascertain the safety and appropriateness of different exercise modalities for glaucoma patients.

Interestingly, the advantageous effects of exercise on IOP reduction appeared more pronounced in patients with higher baseline IOP levels. While no studies were found to address this phenomenon specifically, a more pronounced resting blood pressure reduction was reported in hypertensive patients compared to normotensive ones post-exercise [41].

This review, though innovative in including the highest quality evidence on the effects of physical exercise training programmes on IOP in glaucoma patients, is not without its limitations. Primarily, the meta-analysis could not be performed on studies incorporating an exercise programme due to considerable heterogeneity and a dearth of studies. Additionally, the differences in intensity and duration of activities, as well as the variety of tasks proposed, present challenges in comparing the effect magnitude across interventions. We have also noted that IOP can vary significantly among individuals, with these variations often being more pronounced in patients with glaucoma, adding another layer of complexity to our findings. Lastly, variations in study designs could have contributed to the disparate outcomes. Future research should strive for larger sample sizes, standardised exercise protocols, and longer follow-up periods to yield more reliable conclusions.

In summary, although the evidence remains inconclusive, exercise, particularly aerobic exercise, shows potential in modulating IOP. These practices could serve as complementary therapy in glaucoma patients, potentially reducing glaucoma progression risk. However, the current evidence does not advocate such practices as a substitute for pharmaceutical interventions or other treatment options.
